# Paeoniflorin Inhibits EMT and Angiogenesis in Human Glioblastoma *via* K63-Linked C-Met Polyubiquitination-Dependent Autophagic Degradation

**DOI:** 10.3389/fonc.2022.785345

**Published:** 2022-07-26

**Authors:** Zhi Liu, Zhaotao Wang, Danmin Chen, Xiaorui Liu, Guoyong Yu, Yan Zhang, Chen Chen, Ruxiang Xu, Yezhong Wang, Ru-en Liu

**Affiliations:** ^1^ Department of Neurosurgery, Peking University People’s Hospital, Peking University, Beijing, China; ^2^ Department of Neurosurgery, The Second Affiliated Hospital of Guangzhou Medical University, Guangzhou Medical University, Guangzhou, China; ^3^ Department of Pharmacy, Affiliated Cancer Hospital and Institute of Guangzhou Medical University, Guangzhou, China; ^4^ Affiliated Bayi Brain Hospital, The Seventh Medical Center of PLA General Hospital, Beijing, China

**Keywords:** paeoniflorin, glioblastoma, c-Met, EMT, angiogenesis, polyubiquitination, autophagy

## Abstract

Epithelial-to-mesenchymal transition (EMT) and angiogenesis have emerged as two pivotal events in cancer progression. Paeoniflorin has been widely studied in experimental models and clinical trials for cancer treatment because of its anti-cancer property. However, the underlying mechanisms of paeoniflorin in EMT and angiogenesis in glioblastoma was not fully elucidated. The present study aimed to investigate whether paeoniflorin inhibits EMT and angiogenesis, which involving c-Met suppression, while exploring the potential ways of c-Met degradation. In our study, we found that paeoniflorin inhibited EMT *via* downregulating c-Met signaling in glioblastoma cells. Furthermore, overexpressing c-Met in glioblastoma cells abolished the effects of paeoniflorin on EMT. Moreover, paeoniflorin showed anti-angiogenic effects by suppressing cell proliferation, migration, invasion and tube formation through downregulating c-Met in human umbilical vein endothelial cells (HUVECs). And c-Met overexpression in HUVECs offset the effects of paeoniflorin on angiogenesis. Additionally, paeoniflorin induced autophagy activation involving mTOR/P70S6K/S6 signaling and promoted c-Met autophagic degradation, a process dependent on K63-linked c-Met polyubiquitination. Finally, paeoniflorin suppressed mesenchymal makers (snail, vimentin, N-cadherin) and inhibited angiogenesis *via* the identical mechanism in an orthotopic xenograft mouse model. The *in vitro* and *in vivo* experiments showed that paeoniflorin treatment inhibited EMT, angiogenesis and activated autophagy. What’s more, for the first time, we identified c-Met may be a potential target of paeoniflorin and demonstrated paeoniflorin downregulated c-Met *via* K63-linked c-Met polyubiquitination-dependent autophagic degradation. Collectively, these findings indicated that paeoniflorin inhibits EMT and angiogenesis *via* K63-linked c-Met polyubiquitination-dependent autophagic degradation in human glioblastoma.

## Introduction

Glioblastoma is the most common malignant tumor in the central nervous system and exhibits rapid growth and high invasiveness (1). Over the past few decades, multiple methods of treating the disease, including surgery, chemotherapy, radiotherapy and combination treatments, have been developed; however, patients diagnosed with glioblastoma seldom survive more than 1.5 years (2, 3). Therefore, the identification of new therapeutic agents and the exploration of novel intervention targets may be clinically beneficial for patients undergoing glioblastoma therapy.

Accumulating evidence demonstrates that epithelial-mesenchymal transition (EMT) is related to cancer development and metastasis. Cancer cells with the EMT phenotype have lost their epithelial characteristics and acquired mesenchymal properties *via* upregulation of mesenchymal markers, such as vimentin and snail, which strengthen cell proliferation, migration and invasion capacity and allow cancer cells to invade adjacent tissues and blood vessels and/or to detach from the primary site (4, 5). The role of EMT in glioblastoma is controversial; however, studies exploring the neuro-epithelial theory have acquired evidence showing that an EMT-like process plays a crucial role in initiation and progression of glioblastoma (6). And EMT is induced by a variety of molecules and signaling pathways. Of these, c-Met, an important member of the receptor tyrosine kinase (RTK) family, has been demonstrated to play an important role in EMT regulation (7, 8). And once activated by its unique ligand, HGF, it could regulate many molecules and signaling pathways to facilitate tumor development (9). Moreover, recent studies have shown that higher c-Met expression allowed cancers to acquire more malignant abilities and high levels of c-Met is associated with poor prognosis including glioblastoma (10–12). Therefore, therapies capable of facilitating EMT inhibition and c-Met inactivation may prevent glioblastoma onset and progression and may thus have potential in glioblastoma treatment (13).

Angiogenesis, the process that facilitates the growth of new capillary blood vessels, is a crucial process in the growth, maintenance and metastasis of solid tumors. Angiogenesis is a highly complex process involving endothelial cell proliferation, migration, invasion and tube formation (14). Increasing amounts of evidence indicate that glioblastoma development and metastasis are critically dependent on angiogenesis and that tumor angiogenesis levels are associated with prognosis in patients with glioblastoma. Given the importance of angiogenesis in tumor progression, Dr. Folkman and collogues proposed the concept of “anti-angiogenic therapy” (15, 16). Several successful studies have shown that the inhibition of tumor angiogenesis may contribute to improvements in cancer treatment (17). And it has been reported that angiogenesis could be regulated by c-Met. For example, Ponzo et al. reported that c-Met is expressed in endothelial cells and promotes endothelial cell growth, invasion and motility. Moreover, c-Met could affect angiogenesis by modifying vascular endothelial growth factor (VEGF) (18, 19), which is thought to be an important therapeutic target in glioblastoma. Additionally, the inhibition of c-Met activity impairs the survival, invasion and tube formation of HUVECs *in vitro* and reduces neovascularization and the formation of micro vessels in tumor models (20, 21).Thus, inhibiting c-Met-induced angiogenesis will be an potential approach to treat glioblastoma.

Paeoniflorin, one of the natural compound isolated from Paeonia Lactiflora Pall, has exhibited anti-inflammatory, anti-oxidative, neuroprotective and metabolic regulatory effects (22–24), and increasing numbers of investigations have indicated that paeoniflorin exhibits anticancer effects by inducing apoptosis, restraining proliferation, and suppressing migration and invasion in tumor cells involving various signaling molecular regulation, such as Stat3, TLR4, Norch-1, PI3K/AKT and ERK. In recent, paeoniflorin showed anti-EMT effects in breast cancer and colorectal cancer cells (25, 26).

Our previous studies showed that paeoniflorin inhibited EMT *via* modulating TGFβ in glioblastoma cells (27). Furthermore, paeoniflorin suppressed ox-LDL-induced tube formation *in vitro* and inhibited angiogenesis in chronic hepatic schistosomiasis *in vivo (* 28, 29).Until now, the effects and underling mechanism of paeoniflorin on EMT and angiogenesis in glioblastoma have not been clarified.

Autophagy is a physiological or pathological process that degrades misfolded proteins, damaged organelles for adaptation to starvation, development, cell death, and tumor suppression. Accumulating evidence demonstrated that autophagy promotion could exert anti-cancer effects. Therefore, various studies attempting to find a way to induce autophagy in tumors. Multiple natural compounds, such as curcumin, quercetin, and resveratrol, have been reported that these could inhibit cancers (including glioblastoma) *via* promoting autophagy process (30–32). As a natural compound, paeoniflorin has been shown to regulate autophagy in nerve injury and neurodegenerative diseases (33, 34). However, the effects of paeoniflorin on tumor autophagy remain unclear.

Autophagy-lysosome and ubiquitination-proteasome systems are the two most protein degradation systems and ubiquitination is the link between them. Moreover, difference of ubiquitination modification, such as monoubiquitination and polyubiquitination that includes various lysine residue site ubiquitination, will lead to different fate for the substrate. For example, the monoubiquitination usually mediates protein interactions, locations and trafficking, while the polyubiquitination often involves the protein proteasomal and autophagic degradation. It has been reported that c-Met not only could be modified by monoubiquitin but also polyubiquitin (35). Furthermore, it has showed that c-Met could be degraded through proteasome pathway *via* Lys48-linked polyubiquitin chains (36). Meanwhile, previous study reported c-Met could be degraded in lysosome pathway (37). In our study, we intend to investigate the degradation pathway of c-Met after treatment with paeoniflorin in gliobastoma.

In our present study, we demonstrated for the first time that paeoniflorin inhibited EMT and angiogenesis by downregulating c-Met in glioblastoma *in vitro* and *in vivo*. Furthermore, we showed that paeoniflorin activated autophagy and promoted K63-linked polyubiquitination-dependent c-Met autophagic degradation *in vitro* and *in vivo*. Taken together, our findings show that paeoniflorin has potential as an anticancer agent and serve as evidence showing that c-Met inhibition is the mechanism by which paeoniflorin exerts its effects.

## Method and Materials

### Chemicals, Reagents and Antibodies

Paeoniflorin was purchased from Sigma-Aldrich (St. Louis, MO, USA) and was dissolved in normal saline and stored at 4°C. Dulbecco’s modified Eagle medium (DMEM) and fetal bovine serum (FBS) were purchased from Gibco (Grand Island, USA). Antibodies against c-Met, p-Akt, p-PI3K, N-cadherin, vimentin, p-mTOR, p-P70s6k, p-s6, p62, LC3, ubiquitin (P4D1), and GAPDH were purchased from Cell Signaling Technology (Beverly, MA), and antibodies against VEGF and snail were purchased from Abcam (Cambridge, MA). Antibodies against ubiquitin (FK1) were purchased from Viva Bioscience (Exeter, UK). Chloroquine (CQ), MG132,3-MA, and actidione (CHX) were purchased from Selleckchem (Houston, USA).

### Cell Culture

The human normal astrocytes cell line HEB and HA1800 and the human glioblastoma cell lines U87, U251 and U87-luciferase and HUVECs were purchased from the Chinese Academy of Medical Sciences (Beijing, China). The HEB, HA1800, U87, U251 and U87-luciferase cell lines were cultured in high-glucose DMEM supplemented with 10% FBS, and the HUVECs were cultured in endothelial cell medium (ECM) supplemented with 1% endothelial cell growth supplement (ECGS) and 10% FBS. All cells were incubated at 37°C in a humidified atmosphere of 5% CO2.

### Cell Viability Assay

The cells were seeded in a 96-well plate at a density of 4×10^3^ cells/well for 24 hours and then treated with paeoniflorin. Ten microliters of CCK-8 solution was then added to each well, and the cells were incubated for 1 hour at 37°C. The absorbance of the reaction mixture was subsequently measured by a microplate reader.

### Wound-Healing Assay

A wound-healing assay was used to compare the migratory ability of the cells in the control and experiment groups. The cells (5×10^5^cells) were seeded and cultured in 6-well plates. When the cells reached 80–90% confluence, scratches of a predetermined length were introduced into the monolayers by a sterile pipette tip. The monolayers were rinsed with PBS to remove the detached cells, and then the medium was replaced with medium containing paeoniflorin or normal saline. To distinguish the contributions of cell proliferation to wound closure from those of migration, we treated the cells with the cell cycle blocker hydroxyurea (5 mM, Sigma, Aldrich) at the time of the experiment. To analyze cell migration, we photographed the wounds at the indicated time points with a Leica microscope (Melville, NY). The images were processed using Image Pro-Plus Software (NIH). The wound-healing percentage was determined as follows: [1- (empty area X h/empty area 0 h)]×100.

### Cell Invasion Assay

The transwell system used for the cell invasion assay was obtained from Corning (Corning, USA). The cells (1×10^5^ in 200 μL of DMEM or ECM supplemented with 1% FBS) were seeded in the upper chamber (8 μm), which was coated with 100 µL of Matrigel (BD Biosciences, CA, USA), while 600 μL of DMEM or ECM supplemented with 20% FBS was added to the lower chamber. After 24 hours, the cells in the lower chamber were fixed with methanol and stained with 0.1% crystal violet in methanol. Three independent fields in each well were photographed at 100×, after which the cells in each field were counted.

### Capillary Tube Formation Assay

HUVECs were cultured in a 24-well plate coated with Matrigel (BD Pharmingen, San Diego, CA) at 37°C. After the cells were treated with the indicated concentrations of paeoniflorin for the indicated times, the formation of capillary-like structures was observed under a light microscope. The number of formed tubes, which served as a quantitative index of the degree of angiogenesis *in vitro*, was counted in five low-power fields (100× magnification).

### Chorioallantoic Membrane (CAM) Assay

Fertilized White Leghorn chicken embryos were randomly assigned to two groups comprising five embryos each and were collected into sterile containers for incubation in a humidified environment for 48 hours at 37.5°C on day 3. On day 5, 20 μM paeoniflorin was added to saturation to a microbe-sterilized Whatman filter disk and placed on the CAM *via* the creation of a small hole in the superior surface of the egg. PBS was used as a control. On day 7, the CAM was cut, fixed with acetone and viewed under a microscope. Neovascularization around the disk was quantitated by determining the number of angiogenic vessels around the disk.

### Reverse Transcription Polymerase Chain Reaction (RT-PCR)

Total cellular RNA was extracted using Trizol reagent (Sigma-Aldrich). Equal amounts of first-strand cDNA were synthesized with a FastQuant RT Kit (Tiangen Biotech, Beijing, China), and PCR was performed with Taq DNA polymerase (Takara, Dalian, China) using the following primers: human c-Met: CATCTCAGAACGGTTCATGCC (forward) and TGCACAATCAGGCTACTGGG (reverse); and human GAPDH: TTGGTATCGTGGAAGGACTCA (forward) and TGTCATCATATTTGGCAGGTT (reverse).

### Immunofluorescence

Decapitated brains were immersed in 4% paraformaldehyde for 24 hours for fixation, after which they were placed in 30% sucrose in PBS. Both steps were performed at 4°C. Coronal sections were cut at a thickness of 15 µm in a cryostat. The sections were then washed with PBS containing 0.3% Triton X-100 (PBST) 3 times for 10 minutes each before being blocked with 10% BSA in PBS for 1 h and incubated with the appropriate primary antibodies overnight at 4°C. The sections were subsequently washed with PBS 3 times and incubated with the appropriate Alexa Fluor-conjugated secondary antibodies for 2 hours at room temperature. The sections were then washed with PBS 3 times and mounted in mounting medium containing DAPI (Vector Laboratories, Burlingame).

### Immunohistochemistry

Tissue slides were incubated for 1 hour at 37°C and then deparaffinized. Antigen retrieval was performed by treating the tissues in citrate buffer in a microwave for 10 minutes. After peroxidase activity was blocked with 3% H_2_O_2_/methanol for 10 minutes, the sections were incubated with normal goat serum for 10 minutes to block nonspecific antibody binding. The sections were incubated with the appropriate primary antibodies for 1 hour at 25°C before being incubated with biotinylated anti-rabbit/mouse IgG and peroxidase-labeled streptavidin for 10 minutes each. The images were acquired by a microscope and were analyzed by Image Pro-Plus software.

### Transfection

To overexpress c-Met, we transfected the above cell lines with plasmids carrying c-Met-flag or flag only (GeneCopoeia, Maryland Rockville, USA) using Lipofectamine 3000, according to the manufacturer’s protocol. The same protocol was used to overexpress LC3-GFP in the above cells.

### Western Blotting

Western blotting was performed using cell lysates or xenograft glioblastoma tissue homogenates. Protein was extracted using Pro-prep TM protein Extraction Solution (iNtRON Biotechnology, Korea), according to manufacturer’s instructions. Equal amounts of total protein were separated by 10–12% sodium dodecyl sulfate-polyacrylamide gel electrophoresis (SDS-PAGE) and then transferred to polyvinylidene difluoride membranes (Merck, KGaA, Darmstadt, Germany), which were blocked with 5% BSA for 1 h at room temperature before being incubated with specific primary antibodies overnight at 4 °C. The membranes were then incubated with the appropriate HRP-conjugated secondary antibodies for 1 hour at room temperature. The resulting signals were obtained using Super Signal ECL (Pierce, Rockford, IL, USA).

### Co-Immunoprecipitation

Cells or tumor tissues were lysed with RIPA buffer containing a protease inhibitor cocktail. The lysates were precleared with protein A/G plus agarose beads (Santa Cruz, Dallas) for 2 hours and then incubated with antibody-conjugated beads overnight at 4°C. The beads were washed 3 times with RIPA buffer, re-suspended in SDS electrophoresis sample buffer, and then boiled for 5 minutes at 95°C, after which the samples were subjected to SDS-PAGE and western blot analysis.

### Treatment of the U87 Xenograft Mouse Model With Paeoniflorin

Female BALB/c nude mice were obtained from Vital River Laboratories (Beijing, China). The mice were aged 6 weeks and were maintained in accordance with a standard protocol approved by the Institutional Animal Care Committee of Army General Hospital. All procedures performed in the studies involving the animals were compliant with the ethical standards of the institution or practice at which the studies were conducted. The mice were anesthetized with 3.6% chloral hydrate in 0.9% sterile saline. Each mouse was then intracranially injected with of 4 μL of cultured U87-luciferace cells (1×10^6^ cells per mouse) at a rate of 0.5 μL/min using a Micro 4 Microsyringe Pump Controller (World Precision Instruments, Sarasota, FL) attached to a Hamilton syringe with a 33-gauge needle (Hamilton, Reno, NV). The injection was performed in the mid-right striatum at the following coordinates (in mm from the bregma): +0.5 anterior-posterior, +2.0 medio-lateral, and -2.8 dorso-ventral. After 7 days of cell transplantation, the tumor–bearing mice were distributed into two groups (n=8 each) and intraperitoneally injected with paeoniflorin (400 mg/kg/day) or vehicle (equivalent amount of PBS). Tumor sizes and body weights were measured once every 7 days. At the end of the experiments, the mice were sacrificed, and the tumors were resected and homogenized for western blotting.

### Bioluminescence Imaging

D-luciferin was purchased from Abcam (Cambridge, MA) and was resuspended in PBS at a concentration of 100 mg/mL. The mice were intraperitoneally injected with 150 mg/kg body weight D-luciferin and imaged 10 minutes thereafter using an IVIS^®^ Spectrum optical imaging system fitted with an XGI-8 Gas Anesthesia System (Caliper Life Sciences, Hopkinton, MA). Bioluminescent images were acquired using the auto-exposure function. Analysis of the signal intensities and image comparisons were performed using Living Image^®^ Software (Caliper Life Sciences).

### Statistical Analysis

The data are presented as the mean ± standard deviation from at least three independent experiments. Simple comparisons between two groups were analyzed using independent t-tests, and multiple comparisons between the groups were assessed with one-way ANOVA, followed by *post hoc* analyses, which were performed with the LSD test or Dunnett’s T3 test. All analyses were performed using SPSS 20.0 software. P<0.05 was considered statistically significant.

## Results

### Paeoniflorin Downregulated C-Met and Inhibited HGF-Induced C-Met Activation in Glioblastoma Cells

**Figure 1:**
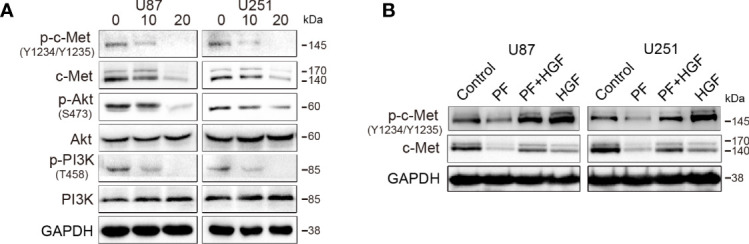
The effects of paeoniflorin on c-Met in the glioblastoma cell lines U87 and U251. **(A)** The cells were incubated with the indicated concentrations (0,10,20 μM) of paeoniflorin for 24 hours, and western blotting was performed to analyze the protein expression levels of c-Met and the downstream signaling molecules. **(B)** The effect of paeoniflorin on HGF-induced p-c-Met activity in U87 and U251 cells. U87 and U251 cells were treated with or without 20 μM paeoniflorin for 24 hours and then incubated with or without 20 nM HGF for 30 minutes. The cells were collected, and western blotting was performed to analyze p-c-Met protein expression levels. All tests were performed in triplicate and the data are presented as the mean ± standard error.

To validate the effect of paeoniflorin on c-Met expression in glioblastoma cells, we treated U87 and U251 cells with different concentrations of paeoniflorin for 24 hours and then performed western blotting to detect c-Met expression, as well as downstream signaling molecule expression. As shown in **Figure 1A**, paeoniflorin downregulated the expression of c-Met and inhibited the activation of p-c-Met and the downstream signaling molecules p-Akt and p-PI3K in a dose-dependent manner in U87 and U251 cells. U87 and U251 cells were incubated with or without 20 μM paeoniflorin for 24 hours before being treated with or without 20 nM HGF, a c-Met-activating ligand. As shown in **Figure 1B**, paeoniflorin successfully suppressed HGF-induced p-c-Met activation. These results suggest that c-Met is a potential target of paeoniflorin in glioblastoma cells.

### C-Met Overexpression Reduced the Effects of Paeoniflorin on Proliferation, Migration, Invasion and EMT in Glioblastoma Cells

**Figure 2:**
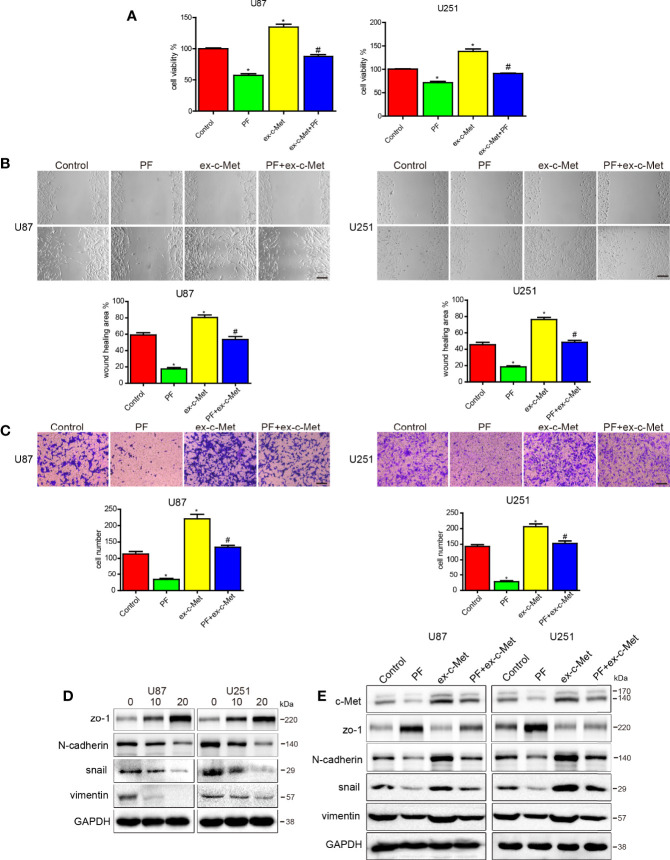
c-Met overexpression reduced the effects of paeoniflorin on cell proliferation, migration and invasion and EMT. **(A)** U87 cells and U251 cells transfected with c-Met or vector plasmids for 12 hours were harvested, and 4×10^3^ cells were seeded in a 96-well plate and then incubated with 10 μM paeoniflorin or PBS for 24 hours. CCK-8 assay was used to detect cell proliferation. **(B)** U87 cells and U251 cells transfected with c-Met or vector plasmids for 12 hours were harvested, and 1×10^5^ cells were seeded in 6-well plates and then incubated with 10 μM paeoniflorin or PBS for 24 hours. Wound-healing assay was performed to detect U87 and U251 cell migration. **(C)** U87 cells and U251 cells transfected with c-Met or vector plasmids for 12 hours were harvested, and 2×10^4^ cells were seeded in a transwell chamber. Transwell assay was performed to evaluate U87 and U251 cell invasion ability. **(D)** U87 and U251 cells were incubated with the indicated concentration of paeoniflorin for 24 hours. Western blotting was performed to examine protein expression. **(E)** U87 and U251 cells were transfected with c-Met or vector plasmids for 12 hours then treated with 20 μM paeoniflorin for 24 hours. Western blotting was performed to examine protein expression. Control: transfected with vector; paeoniflorin: transfected with vector+20 μM paeoniflorin; ex-c-Met: transfected with c-Met; ex-c-Met+paeoniflorin: transfected with c-Met+20 μM paeoniflorin. *P<0.05 vs control. ^#^P<0.05, compared with either paeoniflorin treatment or c-Met transfection alone.

In our previous study, we reported that paeoniflorin inhibited proliferation, migration, invasion and EMT in glioblastoma cells (27, 38) but have no significantly inhibitory effects for the normal astrocyte cell lines HEB and HA1800 in the dose range of 0 to 80μM (**Supplementary Figures 1A, B**). To investigate paeoniflorin exerts these effects whether involve c-Met, we incubated U87 and U251 cells transfected vector or c-Met plasmid with paeoniflorin or vehicle. The results showed that c-Met overexpression significantly decreased the inhibitory effects of paeoniflorin on proliferation in U87 and U251 cells (**Figure 2A**). We also found that c-Met-induced glioblastoma cell migration and invasion were abolished by treatment with paeoniflorin (**Figures 2B, C**). U87 and U251 cells were treated with different doses of paeoniflorin for 24 hours, which resulted in dose-dependent downregulation of the mesenchymal markers N-cadherin, snail and vimentin (**Figure 2D**). However, c-Met overexpression in U87 and U251 cells attenuated the effects of paeoniflorin on c-Met and mesenchymal markers expression (**Figure 2E**). These results suggest that paeoniflorin exerts anticancer effects at least in part by suppressing c-Met-induced EMT in glioblastoma cells.

### Paeoniflorin Inhibited Cell Proliferation, Migration, Invasion and Tube Formation and Downregulated C-Met and VEGF Expression in HUVECs

**Figure 3:**
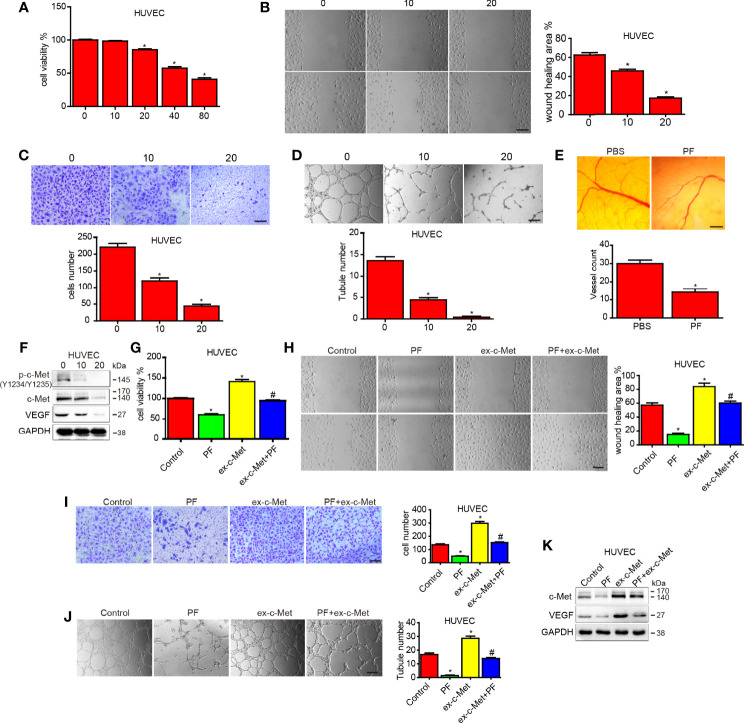
The effect of paeoniflorin on angiogenesis and c-Met overexpression reduced the effects of paeoniflorin on angiogenesis in HUVECs. HUVECs were treated with the indicated concentration of paeoniflorin, and then **(A)** CCK-8 assay was performed to detect cell proliferation. **(B, C)** Wound-healing and transwell assays were performed to detect cell migration and invasion ability, respectively. **(D)** Tube formation assay was performed to evaluate angiogenic ability. **(E)** CAM assay was used to examine the effect of paeoniflorin on angiogenesis. CAMs were treated with 0 or 20 μM paeoniflorin, after which the numbers of micro vessels were counted. **(F)** The effect of paeoniflorin on HGF-induced p-c-Met activity in HUVECs. HUVECs were treated with or without 20 μM paeoniflorin for 24 hours and then incubated with or without 20 nM HGF for 30 minutes. The cells were collected, and western blotting was performed to analyze p-c-Met protein expression. **(G)** HUVECs transfected with c-Met or vector plasmids for 12 hours were harvested, and 4×103 cells were seeded in a 96-well plate and then incubated with 20 mM paeoniflorin or PBS for 24 hours. CCK-8 assay was used to detect cell proliferation **(H)** HUVECs transfected with c-Met or vector plasmids for 12 hours were harvested, and 1×105 cells were seeded in a 6-well plate and then incubated with 20 mM paeoniflorin or PBS for 24 hours. Wound-healing assay was performed to detect cell migration ability.) **(I)** HUVECs transfected with c-Met or vector plasmids for 12 hours were harvested, and 2×104 cells were seeded in a transwell chamber. Transwell assay was then performed to evaluate cell invasion ability. **(J)** HUVECs transfected with c-Met or vector plasmids for 12 hours were harvested, and 1×104 cells were seeded in 24-well plates. Tube formation assay was then performed to evaluate angiogenic ability. **(K)** HUVECs were transfected with c-Met or vector plasmids for 12 hours and then treated with 20 mM paeoniflorin for 24 hours. Western blotting was performed to examine c-Met and VEGF protein expression. Control: transfected with vector; paeoniflorin: transfected with vector+20 mM paeoniflorin; ex-c-Met: transfected with c-Met; ex-c-Met+paeoniflorin: transfected with c-Met+20 mM paeoniflorin. *P<0.05 vs control. ^#^P<0.05, compared with either paeoniflorin treatment or c-Met transfection alone.

To assess the anti-angiogenic properties of paeoniflorin, we used HUVECs to carry out several assays. To investigate the effects of paeoniflorin on HUVEC proliferation, we performed CCK-8 assay. As shown in **Figure 3A**, treatment with paeoniflorin for 24 hours significantly inhibited HUVEC growth in a dose-dependent manner. Cell viability decreased from 83.64% to 36.87% when the concentration of paeoniflorin was increased from 20 μM to 80 μM. We also conducted wound-healing and transwell assays to evaluate the effects of paeoniflorin on HUVECs migration and invasion ability. Low doses of paeoniflorin (10 μM and 20 μM) were used to exclude the effects of paeoniflorin on cell death. The paeoniflorin-treated group exhibited less cell migration into wounds and less cell invasion of the bottoms of insert membranes than the untreated group (**Figures 3B, C**; **Supplementary Figures 1C, D**). These results demonstrated that paeoniflorin significantly inhibited HUVEC migration and invasion. To estimate the effect of paeoniflorin on tube formation, we carried out tube assay and found that treatment with paeoniflorin significantly suppressed or terminated the formation of vessel-like structures. Specifically, paeoniflorin caused the cells to elongate and align in a concentration-dependent manner (**Figure 3D**; **Supplementary Figure 1E**). Since paeoniflorin suppressed angiogenesis *in vitro*, we performed CAM assays to determine whether paeoniflorin represses angiogenesis *in vivo*. Angiogenesis was disrupted in paeoniflorin-treated chicken embryos. This change was signified by the attenuation of micro vessel formation in the CAM and decreases in angiogenic vessel numbers in the paeoniflorin-treated group compared with the PBS-treated group (**Figure 3E**; **Supplementary Figure 1F**). These results suggest that paeoniflorin inhibited angiogenesis *in vitro* and *in vivo*.

We conducted western blotting to examine c-Met and VEGF protein expression in HUVECs treated with different concentrations of paeoniflorin for 24 hours. The results showed that paeoniflorin decreased c-Met and VEGF expression in a dose-dependent manner in HUVECs (**Figure 3F**). 

### C-Met Overexpression Attenuated the Effects of Paeoniflorin on Angiogenesis in HUVECs

To investigate whether paeoniflorin exerts its anti-angiogenic effects by inhibiting c-Met in HUVECs, we treated HUVECs transfected vector or c-Met plasmid with paeoniflorin or vehicle. We found that overexpressing c-Met significantly decreased the inhibitory effects of paeoniflorin on proliferation in HUVECs (**Figure 3G**). We also found that HUVEC migration and invasion were both enhanced by c-Met overexpression but were inhibited by treatment with paeoniflorin (**Figures 3H, I**; **Supplementary Figures 1G, H**). In addition, overexpressing c-Met increased tube formation ability in the HUVECs of the c-Met-overexpression group compared with those of the control group, and treatment with paeoniflorin diminished the effects of c-Met overexpression on HUVEC tube formation ability (**Figure 3J**; **Supplementary Figure 1I**). Moreover, the increases in c-Met and VEGF expression caused by c-Met upregulation were reversed by paeoniflorin in HUVECs (**Figure 3K**). These results suggest that paeoniflorin exerted anti-angiogenic effects in part by downregulating c-Met.

### Paeoniflorin Activated Autophagy in Glioblastoma Cells and HUVECs

**Figure 4:**
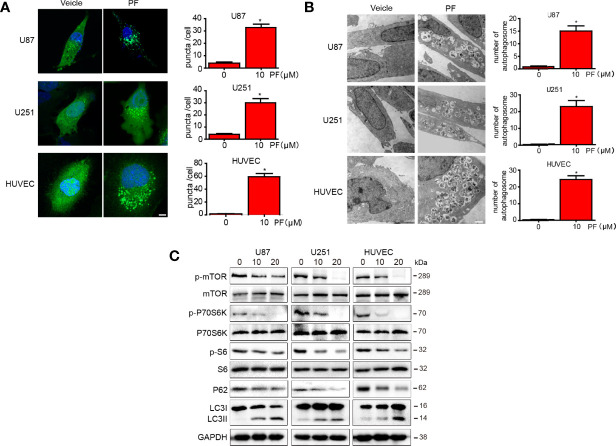
Paeoniflorin induced autophagy in glioblastoma cells and HUVECs. **(A)** U87 and U251 cells and HUVECs transfected with LC3-GFP plasmids for 24 hours were treated with the indicated concentration of paeoniflorin for 12 hours and then imaged, and the numbers of punctate in each cell were counted. Bars, 7.5 μm. **(B)** U87, U251 cells and HUVECs were treated with the indicated concentration of paeoniflorin for 12 hours, and then the cells were harvested before being analyzed by electron microscopy. The number of autophagosomes was counted. Bars, 1 μm. **(C)** U87, U251 cells and HUVECs were treated with the indicated concentration of paeoniflorin for 24 hours, and then p-mTOR, p-P70S6K, p-S6 and LC3 and P62 protein expression was determined by western blotting. All tests were performed in triplicate, and the data are presented as the mean ± standard error. *P<0.05, compared with control (0 µM).

To determine whether paeoniflorin induces autophagy in glioblastoma cells and HUVECs, we incubated U87 and U251 cells and HUVECs transfected with LC3-GFP with the indicated concentration of paeoniflorin for 24 hours. We found that paeoniflorin significantly increased punctate numbers in paeoniflorin-treated U87 and U251 cells and HUVECs compared with PBS-treated cells (**Figure 4A**). To confirm the effects of paeoniflorin on autophagy activation, we used an electron microscope to observe the autophagosome after treating the cells with the indicated concentration paeoniflorin for 24 hours. We found that paeoniflorin treatment significantly increased autophagosome numbers in paeoniflorin-treated U87 and U251 cells and HUVECs compared with PBS-treated cells (**Figure 4B**). To determine the molecular mechanism underlying this phenomenon, we conducted western blotting in U87 and U251 cells and HUVECs treated with paeoniflorin for 24 hours. The results showed that the autophagy-related protein p62 was downregulated, while LC3II was upregulated in paeoniflorin-treated cells compared with PBS-treated cells. Furthermore, we found that paeoniflorin inhibited the mTOR/P70S6K/S6 signaling pathway (**Figure 4C**), a classical autophagy-related pathway. These results indicated that paeoniflorin activated autophagy by inhibiting mTOR/P70S6K/S6 signaling.

### Paeoniflorin Promoted K63-Linked C-Met Polyubiquitination-Dependent Autophagic Degradation in Glioblastoma Cells and HUVECs

**Figure 5:**
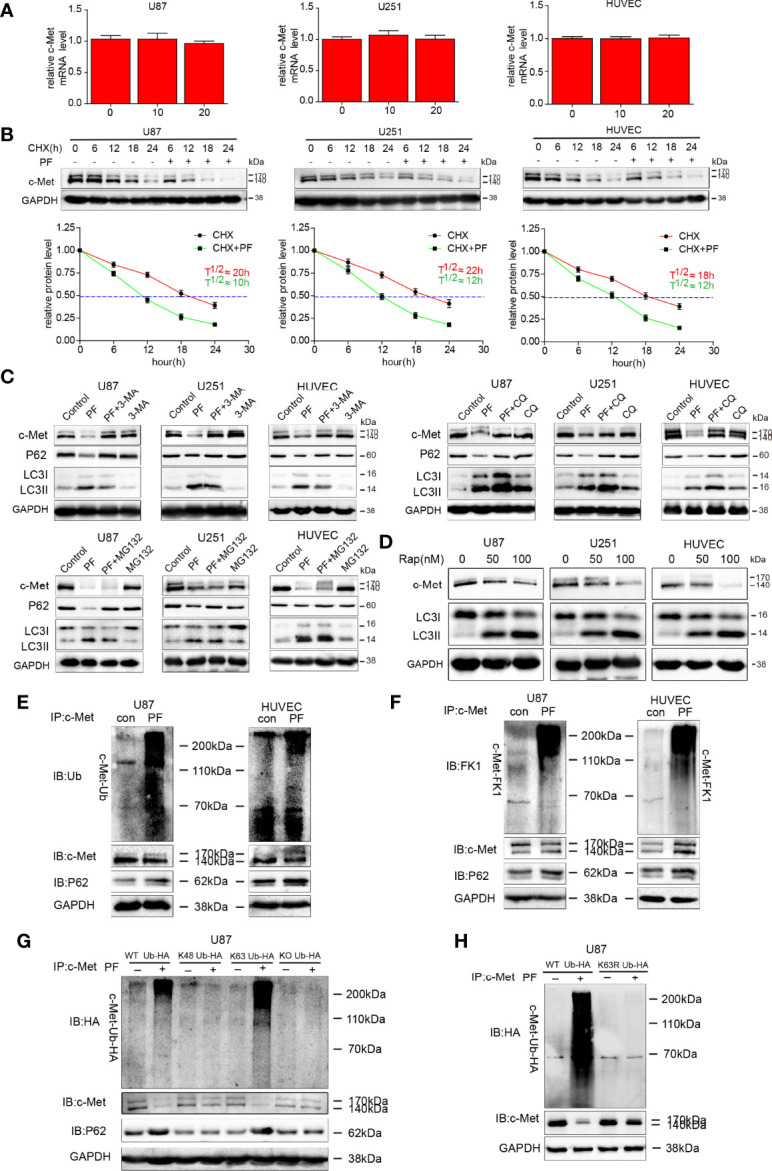
Paeoniflorin promoted K63-linked c-Met polyubiquitination-dependent autophagic degradation in glioblastoma cells and HUVECs. **(A)** c-Met mRNA expression was detected by qRT-PCR in cells treated with different concentrations of paeoniflorin and was normalized to GAPDH expression. Expression was expressed as a fold change relative to 0 μM paeoniflorin-treated U87, U251 cells and HUVECs. **(B)** U87, U251 cells and HUVECs were incubated with 5 mM 3-MA, 20 μM CQ or 5 μM MG132 for 6 hours before being treated with 20 μM paeoniflorin or PBS for 18 hours. c-Met protein expression was estimated by western blotting. **(C)** U87, U251 cells and HUVECs were treated with 50 nM or 100 nM rapamycin for 24 hours. c-Met and LC3 protein expression was assessed by western blotting. **(D)** Time course of c-Met degradation. Top panel, CHX (100 μg/mL) was added to U87, U251 cells and HUVECs treated with or without 20 μM paeoniflorin for 24 hours, after which western blot analysis was performed. Bottom panel, quantified c-Met band intensities, which are representative of three separate analyses by ImageJ (National Institute of Mental Health, Bethesda, MD, USA). The relative intensities of each band from the cell samples were quantified by densitometry as a function of time, with the dotted line (—) indicating the half-life (T½) of c-Met protein in U87 and U251 cells and HUVECs. U87 cells and HUVECs were treated with paeoniflorin (10 μM in U87 cells and 20 μM in HUVECs) for 24 hours. The lysates were subjected to immunoprecipitation, which was performed with antibodies against c-Met, and immunoblot analysis, which was performed with antibodies against **(E)** ubiquitin (P4D1) that recognized monoubiquitin and polyubiquitin and **(F)** antibodies against FK1 that recognized polyubiquitin and p62. U87 cells transfected **(G)** with HA–Ub (WT), HA-UbK48, HA-UbK63 or HA-UbKO or **(H)** with HA–Ub (WT), HA-UbK63R for 12 hours followed by incubating with 20 μM paeoniflorin for 24 hours. The lysates were subjected to immunoprecipitation with antibodies against c-Met and immunoblot analysis.

We first examined c-Met mRNA expression by RT-PCR after the cells were treated with paeoniflorin for 24 hours and found that there was no significant difference in c-Met mRNA expression among U87 and U251 cells and HUVECs (**Figure 5A**). Using CHX, a *de novo* protein synthesis inhibitor, we detected the effect of paeoniflorin on c-Met protein stability. As shown in **Figure 5B**, the half-life of c-Met protein in cells treated with paeoniflorin and CHX was much shorter (approximately 10 hours each in U87 and U251 cells and HUVECs) than that in cells treated with CHX alone (approximately 20 hours in all cells). Thus, c-Met degradation was significantly accelerated (i.e., c-Met stability was dramatically reduced) by treatment with paeoniflorin and CHX. These results indicate that paeoniflorin accelerates c-Met degradation in U87 and U251 cells and HUVECs without interfering with its transcription.

Next, we investigated whether c-Met is degraded through the autophagy-lysosome pathway or the ubiquitin-proteasome pathway by analyzing c-Met protein expression levels in cultured cells treated with paeoniflorin in combination with autophagy-lysosome or ubiquitin-proteasome blockers. The results showed that 3-methyladenine (3-MA), an autophagosome formation blocker, and CQ, a lysosome inhibitor, successfully prevented the degradation of c-Met caused by paeoniflorin; however, MG132, a proteasome blocker, did not inhibit the effects of paeoniflorin on c-Met (**Figure 5C**). These results suggest that paeoniflorin promotes c-Met degradation *via* the autophagy-lysosome pathway. To verify this finding, we treated U87 and U251 cells and HUVECs with rapamycin, an mTOR-dependent autophagy inducer, and found that rapamycin decreased c-Met protein expression levels in a dose-dependent manner in rapamycin-treated cells compared with rapamycin-untreated cells (**Figure 5D**). These results indicate that c-Met was degraded through the autophagosome-lysosome system.

Then we investigated whether c-Met ubiquitination is required for paeoniflorin-induced c-Met autophagic degradation and determined the type of c-Met ubiquitination. We performed co-immunoprecipitation assay with antibodies against c-Met after treating U87 cells and HUVECs with paeoniflorin. We also conducted immunoblot analyses with antibodies against ubiquitin (P4D1) that recognized monoubiquitin as effectively as they recognized polyubiquitin and antibodies against FK1 that recognized polyubiquitin only. We found that degenerated c-Met was detected using antibodies against ubiquitin (P4D1) (**Figure 5E**) and FK1 (**Figure 5F**) and that the intensity of the smeared bands representing c-Met and the binding of p62 to c-Met in paeoniflorin-treated cells was stronger than that in control cells, indicating that c-Met was polyubiquitinated during paeoniflorin-induced degradation.

To identify which lysine site is associated with paeoniflorin-induced polyubiquitination-dependent c-Met degradation, we overexpressed K48 Ub, K63 Ub, WT ubiquitin or knocked out ubiquitin by mutating all the lysine sites into arginine sites in U87 cells and then treated the cells with paeoniflorin. The results showed that the smeared bands representing c-Met and the binding of p62 to c-Met were more intense in cells in which WT or K63 ubiquitin was overexpressed than in other cells (**Figure 5G**). Furthermore, we constructed the K63 mutated ubiquitin plasmid (K63R) and transfected it or the vector plasmid in U87 following paeoniflorin treatment. And we found that the c-Met protein has more degradation only when transfected with vector plasmid, the c-Met protein failed to be degraded when transfected with K63 mutated ubiquitin plasmid under the treatment with paeoniflorin (**Figure 5H**), indicating that paeoniflorin promotes c-Met degradation *via* K63-linked c-Met polyubiquitination.

### Effects of Paeoniflorin on the Orthotopic Xenograft Mouse Model

**Figure 6:**
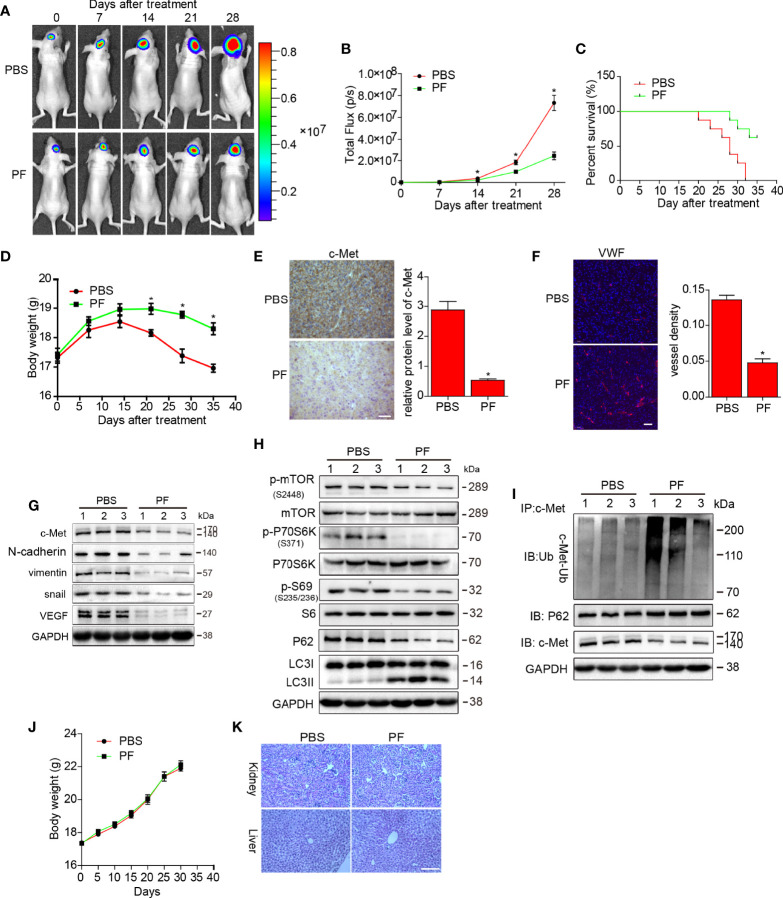
Effects of paeoniflorin on the orthotopic xenograft mouse model. U87-luciferace cells (1×10^6^) were intracranially injected into the mid-right striatum of 6-week-old female BALB/c nude mice. Ten days post-injection, tumor formation was detected by bioluminescence imaging, and the mice were separated into the following two groups: a group comprising mice that were intraperitoneally injected with PPS (control) and a group comprising mice that were intraperitoneally injected with paeoniflorin (400 mg/kg/day). Tumor sizes were measured once every 7 days. Bioluminescence imaging was used to measure tumor volumes. **(A)** A representative tumor volume in each group is shown at each time point **(B)** Tumor volumes were examined at each time point in each group. **(C)** The survival rate of each group. **(D)** Body weight changes in each group. At the end of the experiment or after the mice had died, the brains were excised **(E)** and were stained for c-Met (Bar, 20 μm) and **(F)** VWF (Bar, 50 μm). The images were analyzed by Image-Pro-Plus. At the end of the experiment or after the mice had died, the tumor tissues were excised, and the protein lysates **(G)** were used to estimate c-Met, VEGF and EMT markers expression; **(H)** mTOR/P70S6K/s6 signaling pathway expression; and autophagy-related protein (p62 and LC3) expression by western blotting. **(I)** The lysates were immunoprecipitated with anti-c-Met antibody and immunoblotted with antibodies against c-Met and p62. Normal mice were treated PBS or paeoniflorin (400/kg/day) (for each group n=5) for 30 days **(J)** and the body weights were recorded **(K)** after 30 days, the kidney and liver tissues were obtained and HE-stained (Bar, 500 μm). *P<0.05, compared with control.

We explored the inhibitory effects of paeoniflorin on glioblastoma further in a U87-luciferace orthotopic xenograft mouse model. The results showed that tumor volumes were significantly decreased (**Figures 6A, B**), and mouse survival rates were enhanced (**Figure 6C**) in the 28-day paeoniflorin-treated group compared with the PBS-treated group. Meanwhile, the body weight of mice treated with paeoniflorin were higher than that in PBS-treatment group (**Figure 6D**).

We then detected c-Met protein expression by immunohistochemical staining and found that c-Met expression was reduced in the paeoniflorin-treated group compared with the PBS-treated group (**Figure 6E**). We stained vessels in tumor tissues from U87 xenograft mouse brains and found that the vessel density in the paeoniflorin-treated group was lower than that in the PBS group (**Figure 6F**). Furthermore, the endogenous expression of c-Met, snail, vimentin and VEGF was suppressed in tumor tissues from U87 xenograft mice treated with paeoniflorin compared with tumor tissues from mice treated with PBS (**Figure 6G**). LC3II expression was increased, p62 expression was decreased, and mTOR/p70S6K/S6 signaling was downregulated in the paeoniflorin-treated group compared with the PBS-treated group (**Figure 6H**), indicating that paeoniflorin inhibited EMT and angiogenesis and induced autophagy in the orthotopic xenograft mouse model. We also performed co-immunoprecipitation in cells from the above tumors and found that paeoniflorin promoted c-Met polyubiquitination-dependent autophagosome degradation in the intracranial xenograft mouse model (**Figure 6I**). Our *in vivo* study results were consistent with the *in vitro* results demonstrating that paeoniflorin plays a critical role in the suppression of glioblastoma growth in an intracranial tumor model by inhibiting c-Met-mediated EMT and angiogenesis.

To evaluate the safety of paeoniflorin *in vivo*, we treated normal mice with PBS or paeoniflorin (400/kg/day) (for each group n=5) for 30 days and recoded their body weights every 5 days. We then obtained kidney and liver tissues from PBS- and paeoniflorin-treated mice and conducted HE staining. As shown in **Figure 6J**, body weight did not differ significantly between the two groups. Moreover, we noted no clear pathological changes in the kidney and liver tissues of the two groups (**Figure 6K**). These findings suggest that paeoniflorin is safe when administered *in vivo*.

## Discussion

In our present study, we noted that paeoniflorin suppressed c-Met-mediated EMT and angiogenesis in glioblastoma cells. Additionally, we observed that paeoniflorin induced autophagy activation. Furthermore, we showed for the first time that paeoniflorin degraded c-Met in an autophagy-lysosome-dependent manner though a process related to K63-linked c-Met polyubiquitination. Finally, we demonstrated that paeoniflorin suppressed glioblastoma in an intracranial glioblastoma mouse model and that the drug acted through the same mechanism *in vivo* as *in vitro*.

c-Met, as a known EMT and angiogenesis modulator, we for the first time identified it as a potential target of paeoniflorin. Recent investigations have reported that paeoniflorin acts as important regulators in EMT. For instance, paeoniflorin suppresses TGF-β mediated EMT in pulmonary fibrosis through a Smad-dependent pathway (39). Additionally, other studies reported that paeoniflorin also inhibited EMT in human colorectal cancer cells, glioma cells and breast cancer cells (25–27). However, the relation between paeoniflorin, EMT and c-Met pathway is not clear in glioblastoma cells. In this study, we showed that paeoniflorin inhibited EMT process through downregulating the mesenchymal markers like N-cadherin, snail and vimentin, which has been reported highly expressed in glioma and were closely related to the poor prognosis (40). Meanwhile, PF upregulated the epithelial marker zo-1, which has been reported that it is low expressed in glioma and its suppression could inhibit the invasion ability in glioma cells (41, 42). And we consummated the mechanism and demonstrated that PF exerted anti-EMT effects by downregulating c-Met. Agents targeting angiogenesis continue to be explored to inhibit cancers (43, 44). In our present study, we demonstrated that paeoniflorin inhibited HUVEC proliferation, migration, invasion and tube formation ability, an effect that was associated with c-Met suppression. Moreover, we confirmed the anti-angiogenesis ability of paeoniflorin by CAM assay. Furthermore, at the molecular level, paeoniflorin inhibited the expression of VEGF, the most important molecule in angiogenesis, *in vitro* and *in vivo*. These are consistent with the previous study that paeoniflorin inhibited tube formation in HUVECs cells *via* VEGF and notch pathways and paeoniflorin suppressed angiogenesis in experimental BALB/c mansoniasis model (45). However, Yuan R et al. reported that paeoniflorin enhanced angiogenesis in a vascular insufficiency model of zebrafish *in vivo* and in HUVECs *in vitro*. For the disparity, one reason may be the difference of the model, after all, the reaction of mammalian and fish to a drug sometimes are very different. Another reason is the concentration of paeoniflorin we used, Yuan R et al. applied a less dose than us. In this study, we for the first time reported that paeoniflorin could suppressed angiogenesis in glioblastoma model, which suggests paeoniflorin may be a latent anti-angiogenesis agent to treat glioblastoma.

Autophagy, an important posttranscriptional process responsible for degrading proteins, has been reported to affect cancer initiation and progression, and a larger number of studies have shown that autophagy activation suppresses cancer cell proliferation, invasion, and EMT and tumor angiogenesis in cancer (46, 47). Tohnai G et al. found that paeoniflorin enhances autophagy systems in an SBMA model (33). Moreover, Cai and Sun reported that paeoniflorin enhanced autophagy to degrade α-synuclein (48, 49). In our study, we found paeoniflorin strengthened autophagy in glioblastoma cells and HUVECs and the process is likely to involve autophagy because paeoniflorin not only increased the puncta (**Figure 5A**) but also upregulated LC3II. Even if compared with the only paeoniflorin treatment group, the LC3II was also increased in treatment paeoniflorin combined with chloroquine (**Figure 2C**). And this process involved the mTOR/p70s6k/s6 signaling pathway (**Figure 5C**). In present study, we are the first to demonstrate that paeoniflorin promote autophagy in glioblastoma. And glioblastoma suppression may be attributable to EMT and angiogenesis inhibition facilitated by autophagy enhancements.

It has been reported that c-Met can be degraded by both the lysosome system and the ubiquitination-proteasome system (35, 37). Young Mi Oh et al. reported that SAIT301 induces c-Met degradation in the lysosome (50). Stephanie Carter et al. showed that K48-linked c-Met polyubiquitination is important in proteasomal degradation (36). Additionally, Rachel Barrow-McGee et al. showed that c-Met degradation was dependent on a non-canonical autophagy pathway (51). In current study, we demonstrated that paeoniflorin enhanced c-Met polyubiquitination and we identified K63 site dependent polyubiquitination involving the c-Met degradation in autophagy-lysosome pathway. This is the first time to verify that a natural product can degrade c-Met in an autophagy-lysosome pathway.

According to data from the Food and Drug Administration (FDA), approximately 25–48% of currently approved anticancer agents come from plants (52). Paeoniflorin, a monomeric natural compound extracted from Radix Paeonia Alba, has shown anticancer activity in some types of cancer. However, the agents that have been shown to be effective in the subcutaneous tumor model may not effective in the intracranial tumor model due to an inability to penetrate the blood-brain barrier (BBB). Paeoniflorin has the ability to cross the BBB. He et al. found that paeoniflorin quickly penetrated the BBB to reach the hippocampus and that the concentration of the drug remained high (53). Similarly, Cao et al. reported that paeoniflorin penetrated the BBB and reached the normal cortex at a concentration enabling the effective treatment of ischemia-reperfusion in rats (54). In the present study, we demonstrated that paeoniflorin exerted potent anti-glioblastoma effects in an *in situ* glioblastoma model. Moreover, we found that the mouse is tolerant to continuous paeoniflorin administration and does not experience important organ injury during paeoniflorin treatment; thus, paeoniflorin is safe *in vivo*.

In conclusion, we found that paeoniflorin acts as an inhibitor of glioblastoma EMT and angiogenesis *in vitro* and vivo. Moreover, we determined that c-Met may be the target of paeoniflorin in glioblastoma. Additionally, we demonstrated that paeoniflorin induces autophagy activation and that paeoniflorin degraded c-Met *via* the autophagic pathway though K63-linked polyubiquitination.

## Data Availability Statement

The datasets presented in this study can be found in online repositories. The names of the repository/repositories and accession number(s) can be found in the article/**Supplementary Material**.

## Ethics Statement

The animal study was reviewed and approved by the Institutional Animal Care Committee of Army General Hospital.

## Author Contributions

ZL and ZW conducted most of the corresponding cell and molecular biology experiments. GY finished the *in vivo* assay. ZW, YZ, DC, CC and RX made a contribution to draft the manuscript and revision. XL performed statistical analysis. YW and R-EL conceived the subject and guided the writing of the article. All authors contributed to the article and approved the submitted version.

## Funding

This work was supported by the National Natural Science Foundation of China (Grant NO. 82074174)

## Conflict of Interest

The authors declare that the research was conducted in the absence of any commercial or financial relationships that could be construed as a potential conflict of interest.

## Publisher’s Note

All claims expressed in this article are solely those of the authors and do not necessarily represent those of their affiliated organizations, or those of the publisher, the editors and the reviewers. Any product that may be evaluated in this article, or claim that may be made by its manufacturer, is not guaranteed or endorsed by the publisher.

## Supplementary Material

Supplementary Figure 1 The effects of paeoniflorin on the normal astrocyte cell lines in proliferation 4×10^3^ cells **(A)** HEB and **(B) **HA1800 were seeded in a 96-well plate and then incubated with 0-80μM paeoniflorin for 24 hours. CCK-8 assay was used to detect cell proliferation.
