# Supplementary figures and images for: Paeoniflorin Inhibits EMT and Angiogenesis in Human Glioblastoma via K63-Linked C-Met Polyubiquitination-Dependent Autophagic Degradation
Source: Front Oncol. 2022 Jul 26;12:785345. doi: 10.3389/fonc.2022.785345 (PMC9360619; doi:10.3389/fonc.2022.785345)

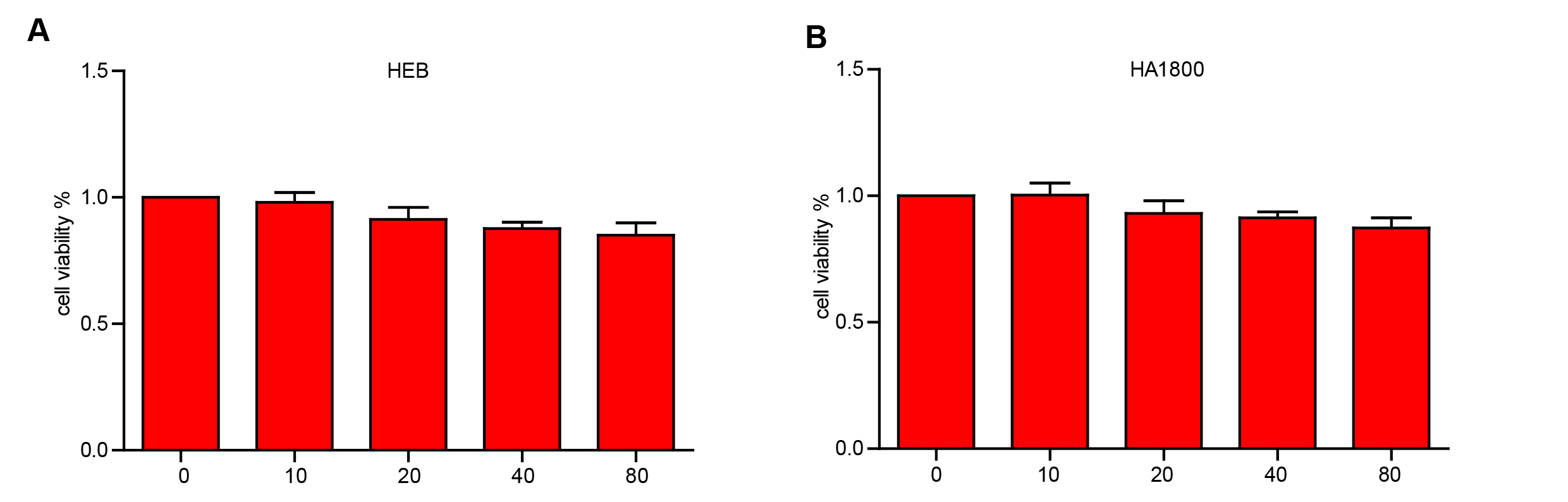

Supplement: Supplementary Figure 1 — The effects of paeoniflorin on the normal astrocyte cell lines in proliferation 4×10^3^ cells (A) HEB and (B) HA1800 were seeded in a 96-well plate and then incubated with 0-80μM paeoniflorin for 24 hours. CCK-8 assay was used to detect cell proliferation. [file Image_1.tif]
